# Quantitative modeling of dose–response and drug combination based on pathway network

**DOI:** 10.1186/s13321-015-0066-6

**Published:** 2015-05-16

**Authors:** Jiangyong Gu, Xinzhuang Zhang, Yimin Ma, Na Li, Fang Luo, Liang Cao, Zhenzhong Wang, Gu Yuan, Lirong Chen, Wei Xiao, Xiaojie Xu

**Affiliations:** Beijing National Laboratory for Molecular Sciences (BNLMS), State Key Laboratory of Rare Earth Materials Chemistry and Applications, College of Chemistry and Molecular Engineering, Peking University, Beijing, 100871 People’s Republic of China; National Key Laboratory of Pharmaceutical New Technology for Chinese Medicine, Kanion Pharmaceutical Corporation, Lianyungang City, 222002 People’s Republic of China

**Keywords:** Dose–response modeling, Drug combination, LPS-induced PGE2 production, Pathway network

## Abstract

**Background:**

Quantitative description of dose–response of a drug for complex systems is essential for treatment of diseases and drug discovery. Given the growth of large-scale biological data obtained by multi-level assays, computational modeling has become an important approach to understand the mechanism of drug action. However, due to complicated interactions between drugs and cellular targets, the prediction of drug efficacy is a challenge, especially for complex systems. And the biological systems can be regarded as networks, where nodes represent molecular entities (DNA, RNA, protein and small compound) and processes, edges represent the relationships between nodes. Thus we combine biological pathway-based network modeling and molecular docking to evaluate drug efficacy.

**Results:**

Network efficiency (*NE*) and network flux (*NF*) are both global measures of the network connectivity. In this work, we used *NE* and *NF* to quantitatively evaluate the inhibitory effects of compounds against the lipopolysaccharide-induced production of prostaglandin E2. The edge values of the pathway network of this biological process were reset according to the Michaelis-Menten equation, which used the binding constant and drug concentration to determine the degree of inhibition of the target protein in the pathway. The combination of *NE* and *NF* was adopted to evaluate the inhibitory effects. The dose–response curve was sigmoid and the EC50 values of 5 compounds were in good agreement with experimental results (R^2^ = 0.93). Moreover, we found that 2 drugs produced maximal synergism when they were combined according to the ratio between each EC50.

**Conclusions:**

This quantitative model has the ability to predict the dose–response relationships of single drug and drug combination in the context of the pathway network of biological process. These findings are valuable for the evaluation of drug efficacy and thus provide an effective approach for pathway network-based drug discovery.

## Background

The dose–response relation is a key topic in pharmacology. How to predict the efficacy of a compound for a system (protein, biological process, cell, tissue, organ and the body) is critical for drug discovery. The drugs (magic bullets) developed in the past decades were designed to selectively target a specific protein. However, when a single drug is administered and enters the body, interaction with 1 or more cellular targets is possible [1, 2]. A drug may produce multiple effects in the system through interacting with multiple cellular targets, which is called “polypharmacology” [2, 3]. The pathogenesis of complex diseases such as cardiovascular disorders and diabetes is related to a lot of genetic and environmental factors [4, 5]. The human body is a complicated, integrated and networked biological system. And drugs which selectively target 1 protein cannot treat complex diseases effectively due to the robustness and redundancy of the biological system [6–11]. Meanwhile, multi-target drug therapies may be more effective than individual high-affinity drugs for complex diseases [12]. Nevertheless, the more drugs administrated or more targets with which drugs can interact, the more complicated the mechanism would be. Therefore, the prediction of drug efficacy is a challenge, especially for complex systems.

When 2 or more drugs are administrated in combination, the interactions among drugs would add a further complication to the prediction of the dose–response relation of drug combination. Generally, the drug interaction would generally produce 1 of 3 different effects: synergism, antagonism and additive effect [13, 14]. Synergism means that drug combination could produce exaggerated effect, and antagonism could reduce the total effect. Synergism is especially important in clinical applications since it allows the use of smaller amounts of drugs and thus reduces the adverse effect or toxicity [14–17].

We have developed a pathway network-based approach to evaluate the efficacy of a compound against biological processes, such as blood clotting [18] and platelet aggregation [5]. Recently, we used this method for virtual screening of active compounds for the inhibition of lipopolysaccharide (LPS)-induced prostaglandin E2 (PGE2) production [19]. In this work, we demonstrate an advance in the quantitative modeling of dose–response and drug combination based on the pathway network of LPS-induced PGE2 production. PGE2 is the principal inflammation mediator, which could participate in many pathological processes [20–22]. The production of PGE2 can be regarded as a biomarker of inflammation. Generally, the pathway of LPS-induced PGE2 production was modeled as a network. And the binding affinity of a compound to a protein in the pathway network was assessed by molecular docking. The docking results had influence on the edge weights by relating them to enzyme efficiency *via* Michaelis-Menten kinetics. The effect of a compound on the entire network, and thus, in this particular case, the production of PGE2, was assessed by using the network connectivity measures (network efficiency and network flux). By integrating molecular docking and network analysis, network efficiency, network flux and their combination can quantitatively describe the inhibitory effects of compounds. Moreover, the efficacy and synergism or antagonism of the combination between 2 compounds were also evaluated.

## Results

### Pathway network of LPS-induced PGE2 production

**Fig. 1:**
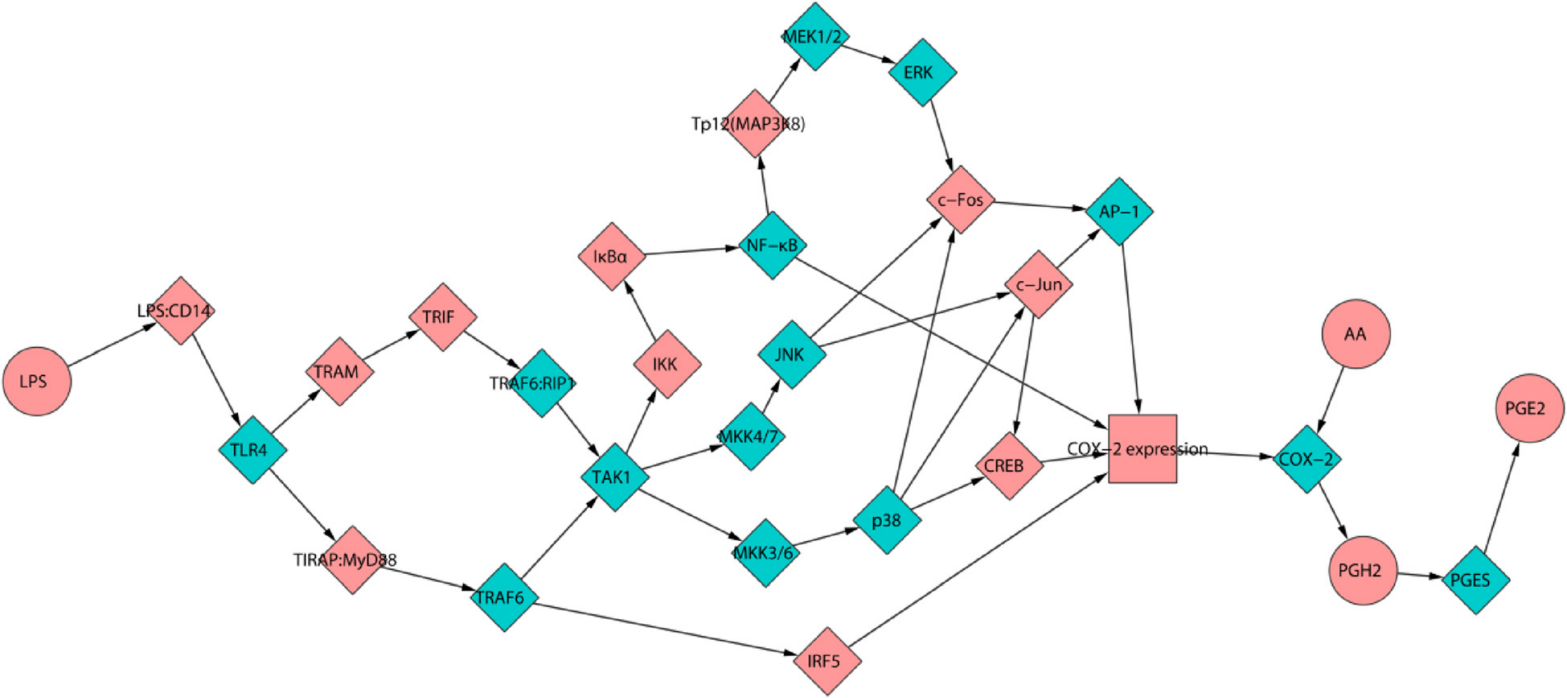
The pathway network of LPS-induced PGE2 production. Circle and diamond represent small molecule and protein, respectively. The target proteins for molecular docking are marked as green diamonds

The pathway network of LPS-induced PGE2 production (Fig. 1) comprised 30 nodes and 38 edges (arrows), where nodes represented proteins and small molecules involved in the process of LPS-induced PGE2 production, and edges meant that the node in front of the arrow was downstream in the pathway. This network was a scale-free and small-world network, which were 2 typical characteristics of biological networks [23]. It indicated that the network can have strong stability and can resist random attacks [23, 24]. However, it would be vulnerable for targeted attacks, such as selective drugs. Therefore, it offered an opportunity for us to develop drugs to treat inflammation-related diseases, especially multi-target drugs and drug combination to simultaneously block multiple targets with varying degrees.

### Network efficiency and network flux

Network efficiency was first proposed by Latora V. and Nagurney A. to measure the importance of a node in a network when the node was removed [25, 26]. Then it was adopted by our lab to evaluate the efficacy of a drug against blood clotting cascade [18] and platelet aggregation [5]. *NE* was a global measure of the connectivity of a network and can reflect the integrity of the network. *NE* was defined as the sum of the shortest path lengths between each node in the network. Thus in the calculation of *NE*, all shortest paths between a node and other N-1 nodes counted (N was the number of nodes in the network). However, it cannot reflect the different weightiness of the node because all nodes were calculated N-1 times [5]. Actually the further downstream a node located in the pathway network, the more important it would be. Thus network flux was proposed to calculate the shortest paths between all other nodes and the exit node of the pathway network in our previous work [5]. The combination of *NE* and *NF* took into account the different importance of the node, thus it can be used to predict the potencies of compounds against platelet aggregation [5].

**Fig. 2:**
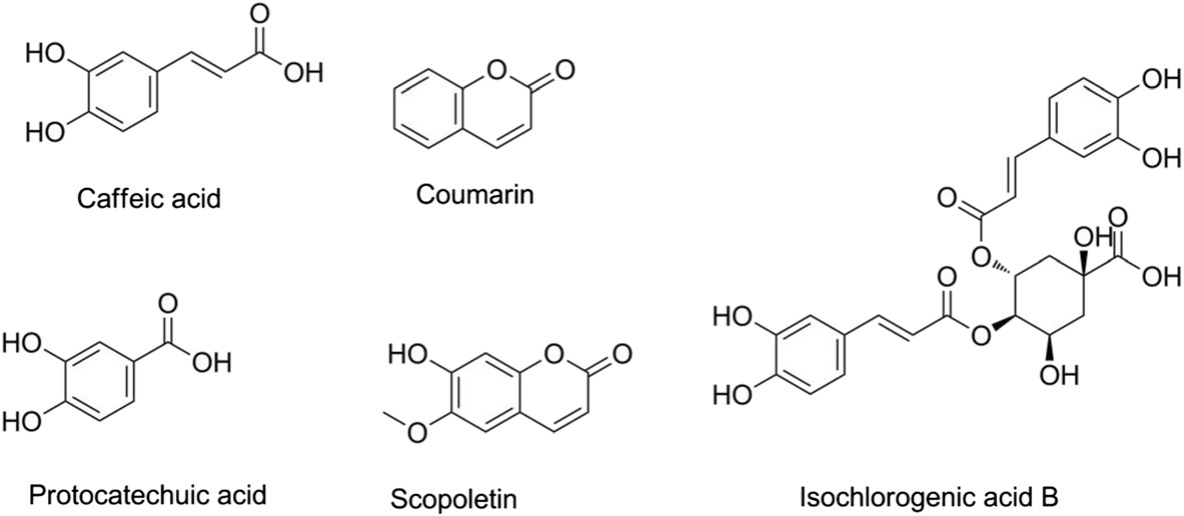
Structures of 5 active compounds

**Fig. 3:**
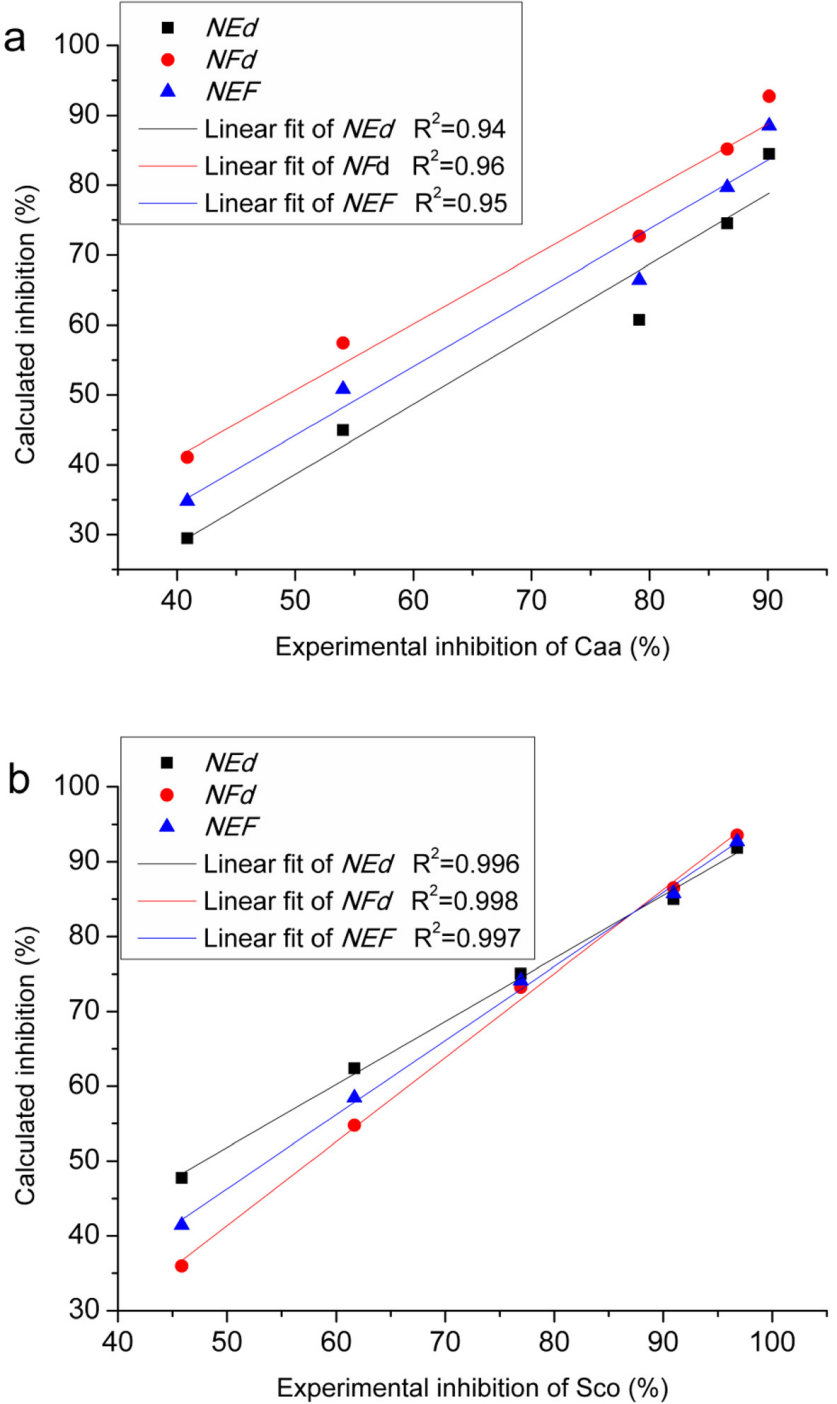
The linear fitting between predicted efficacy and *in vitro* experimental results. (**a**) Caffeic acid; (**b**) Scopoletin. The black square, red dot and blue triangle represent the predictions of *NEd*, *NFd* and *NEF*, respectively

The degree of decrease of *NE* (*NEd*) and *NF* (*NFd*), and the geometric mean of *NEd* and *NFd* (*NEF*) were all indicators of the network connectivity. In this work, we studied 5 active compounds (Fig. 2) extracted from Reduning Injection which was a widely used Chinese medicine prescription [19]. The activities of 5 compounds against LPS induced PGE2 production at different concentrations were predicted. However, *NEd*, *NFd* and *NEF* had different accuracies for the predictions, as shown in Fig. 3. Caffeic acid and Scopoletin were the 2 of the most potent compounds, so the 2 compounds were picked out as examples. In the case of Caffeic acid (Caa), the predictions of *NEd* were lower than the *in vitro* experimental results, while the predicted inhibition rates by *NFd* were higher than experimental results (Fig. 3a). It was more complicated for Scopoletin (Sco): the model had higher predictions at low concentrations and lower predictions at high concentrations by *NEd*, while it was the direct opposite of predictions by *NFd* (Fig. 3b). However, the model gave good agreements between predictions by *NEF* and experiment results in all cases. Therefore, *NEF* was a better evaluation indicator for this system and was used in further evaluations.

### Dose–response curve

**Fig. 4:**
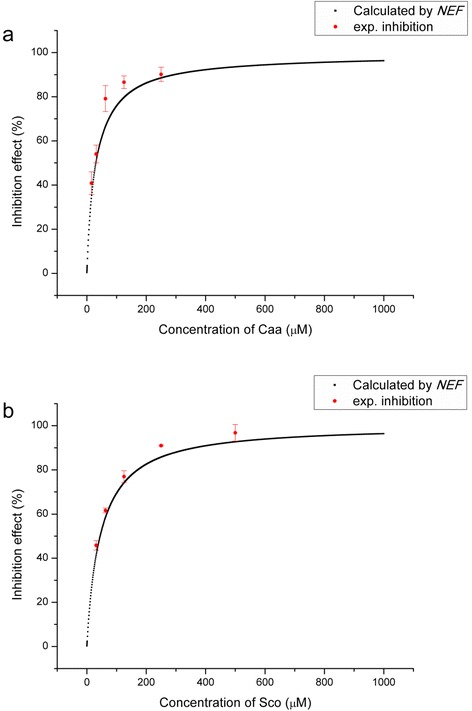
Dose–response curve. (**a**) Caffeic acid; (**b**) Scopoletin

The shape of the dose–response curve was important to evaluate the efficacy of a compound. All predicted and experimental dose–response curves were sigmoid. Table 1 listed the parameters of fitted dose–response curves of 5 active compounds according to the predictions by *NEF*. The E_max_ and E_min_ were close to 100 % and 0, respectively. And the correlation coefficients were higher than 0.999, especially for Caa and Sco (Figs. 4a, b). Moreover, the predicted EC50 values of 5 active compounds well matched with the experiment values (R^2^ = 0.93, Table 1), which indicated that the prediction model by *NEF* would be reliable.

**Table 1:** Parameters of fitted dose–response curves of predictions by *NEF*

Compounds	E_max_ (%)^a^	E_min_ (%)^a^	EC50 (μM)^b^	n	R^2^	EC50e (μM)^b^
Caffeic acid	99.49	0.28	30.20	0.98	0.99994	17.35
Coumarin	99.99	0.85	52.95	1.12	0.99996	49.14
Isochlorogenic acid B	111.20	−3.63	116.16	0.56	0.9998	96.82
Protocatechuic acid	101.18	−1.46	42.82	0.77	0.99995	46.34
Scopoletin	100.72	0.60	45.48	1.01	0.99994	38.46

^a^E_max_ and E_min_ were the top and bottom asymptotes of the response, respectively. ^b^EC50 and EC50e were the concentration of inhibitor at half-maximal effect calculated by predictions and experimental results, respectively

### Drug combination and dose–response surface

**Fig. 5:**
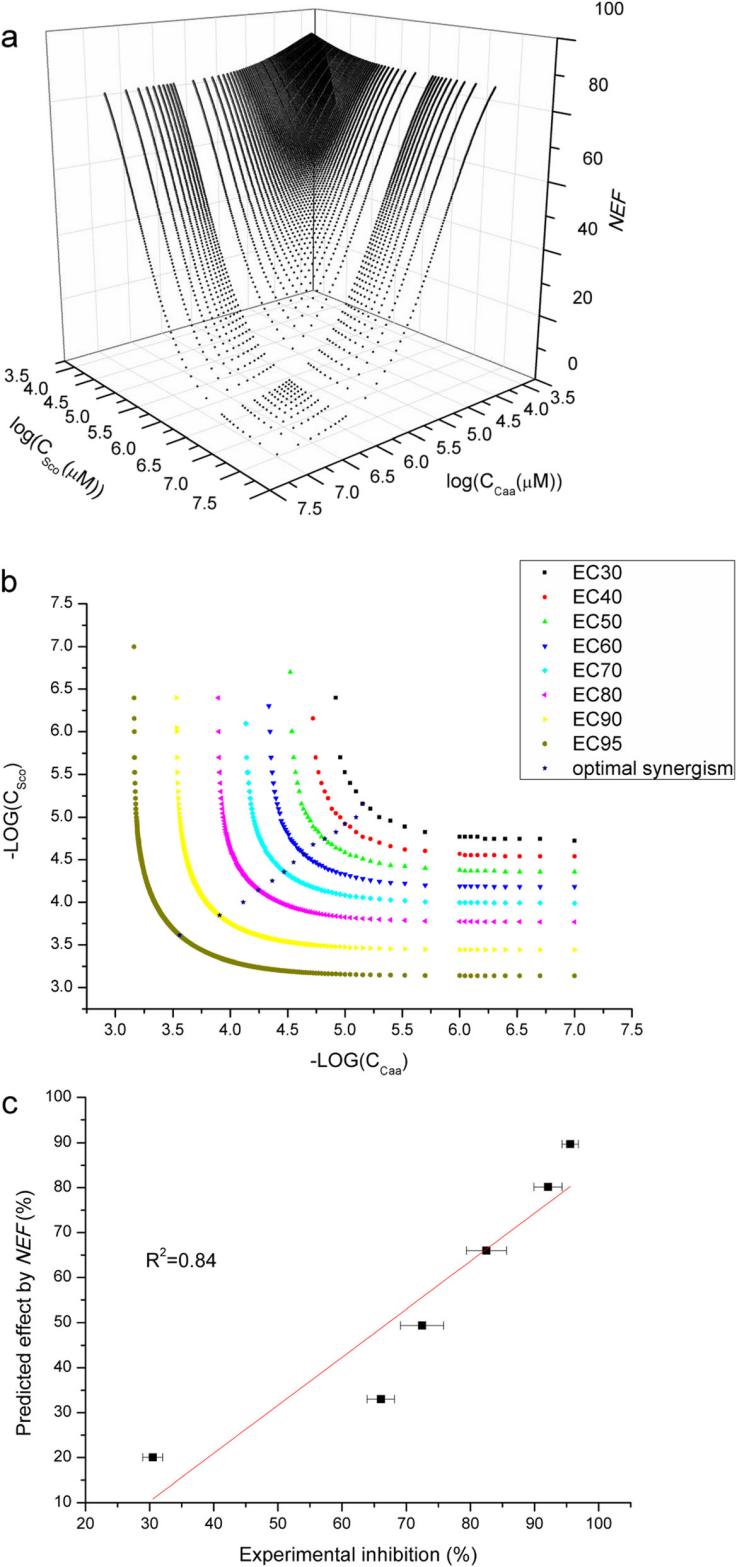
Drug combination. The dose–response surface (**a**) and isobologram (**b**). (**c**) was the comparison between predicted efficacy and experimental inhibition

This approach can also evaluate the combination of 2 or more compounds. There would be 3 effects for drug combination: synergism, additive effect and antagonism. The *combination index* (*CI*) proposed by Chou T. C. was adopted to quantify the synergic degree of drug combination [13]. When 2 drugs both existed in the system, it would produce a dose–response surface (DRS). Fig. 5a showed the DRS of the combinations between Caa and Sco in different doses. A series of drug combination can have the same effects on the system, which can be described by the isobologram (Fig. 5b). Each dose pairs on the isobologram represented possible combinations that produced the equivalent effect. In the case of combination of Caa and Sco, it was a typical synergistic effect. However, each drug pair in the isobologram differed in degree of synergism. The stars in Fig. 5b pointed out the optimal combination for each degree of inhibition. And we found that the dose ratios of 2 compounds for maximal synergism were nearly the same with the ratio between each EC50. Therefore, the experimental inhibition rates of 6 combinations of Caa and Sco with the ratio between each EC50 value were determined. And the results agreed well with the predictions (R^2^ = 0.84, Fig. 5c).

## Discussion

The pathway of a biological process is a minimal biosystem with a specific function and can be abstracted as a network. The network properties can relate with the state of the biosystem to a certain extent, especially for biomarkers related to diseases [5]. Thus the influence on the pathway network of a compound can be used to evaluate the efficacy [5, 18, 23, 27–29]. These results demonstrated that the degree of the decreases of *NE*, *NF* and *NEF* were measures of inhibition of a drug against LPS-induced PGE2 production. A drug could target multiple proteins in the biological pathway, and a drug combination could produce synergistic or antagonistic effect in different extent through multi-target interactions [14, 19, 30]. Synergism is useful in illuminating mechanisms of drug action and exploring computational models to predict new drug leads in drug discovery. The dose–response surface and isobologram are 2 practical tools. Moreover, the *combination index* is a convenience for researchers to determine whether synergism, additive effect, or antagonism exists in a drug combination.

The exact mechanisms of the inhibitions of drugs against LPS-induced PGE2 production are unclear now since the biological system of the cells or organism is complicated. Our recent works indicated that most active compounds would have polypharmacology according to drug-target network [30]. However, the computational approach in this work generally does not need to know about the exact mechanism, which could broaden the scope of application, especially for complex systems.

Although in the above text the predictions of this model agreed well with the experimental results, it would be necessary to note that this approach should require several conditions to obtain reasonable predictions. First, the pathway of a biological process should be as fully accurately as possible. The calculations of *NE* and *NF* are heavily dependent on the completeness of the pathway network. The information of the pathway of LPS induced PGE2 production is abundant and this system has been reviewed in literatures and databases (described in the section of Construction of the pathway network of LPS-induced PGE2 production). However, when others apply this approach in this work to other systems, they need to be very careful. When a pathway has low completeness or lacks sufficient annotations, the predictions would have large deviations. Second, the structures (determined by X-ray or NMR) of most target proteins in the pathway should be known. It's best to obtain ligand-protein complex structure and thus the binding site can be defined as the space which was occupied by the ligand. Beyond that, the binding energy calculated by molecular docking or molecular dynamics simulation should be accurate. The binding predictions from molecular docking are subject to a margin of error due to the principle and method of the calculation. However, we can try to reduced the error. For example, we used the most commonly used software AutoDock4 and adopted the validated protocols whose predictions had been validated by experimental results in our previous works [5, 18]. Big errors of binding predictions may affect the calculation of edge values and then reduce the accuracies of the calculation of *NE* and *NF*. Furthermore the predictions of inhibition rates of drugs would have big errors. Finally, the prediction model should be validated by experiments. The known active compounds can be used as training set to adjust the parameters of the predicting model.

## Methods

### Construction of the pathway network of LPS-induced PGE2 production

The pathway network of LPS-induced PGE2 production was constructed in our recent work [19] according to the information extracted from KEGG pathway database [31], Reactome [32], and literatures [33–40]. Generally, LPS-induced PGE2 production was involved in 2 pathways: Toll-like receptor signaling pathway (ID: map04620 in KEGG pathway database) and NF-kappa B signaling pathway (map04064). First, LPS can interact with CD14 and the complex facilitates the recognition of LPS stimulation by TLR4. Then the signaling is divided into MyD88-dependent and TRIF-dependent pathways. MyD88 and TRIF can activate transcription factors such as IRF-5, NF-kappa B and AP-1 in the downstream pathway. In particular, Eliopoulos A. G. and colleagues contributed the pathway of CREB, a key regulator of COX-2 transcription [39]. Finally, the pathway comprised 30 nodes and 38 edges (Fig. 1). Cytoscape 2.8 was used to visualize the network and calculate the network properties by Network Analysis plugin [41].

### Molecular docking

There were 14 important proteins (Table 2) which can be used for molecular docking. When a protein had multiple X-ray or NMR structures in RCSB Protein Data Bank (http://www.rcsb.org/pdb/home/home.do), there were several criteria to choose the most suitable structure. First, the structure had more complete peptide chains. Second, the resolution of the structure should be as high as possible. Third, it’s better that the structure had a ligand. The X-ray or NMR structures were downloaded from RCSB Protein Data Bank and treated to suitable for molecular docking by Autodock4 [42, 43] according to the protocols described in previous works [5, 19, 44]. The energy grid was a 20 × 20 × 20 Å cube centered on the occupied space of the original ligand with a spacing of 0.375 Å between the grid points. The maximum number of energy evaluations was set to 2.5 × 10^7^. The AD4score function was used to evaluate the affinity between compound and protein, and the docking score was p*K*_*i*_.

**Table 2:** 14 target proteins for molecular docking

Target	Protein name	UniProt ID	PDB ID
TLR4	toll-like receptor 4	O00206	4G8A
PGES	Prostaglandin E synthase	O14684	3DWW
TAK1	MAP3K7	O43318	2YIY
AP-1	Transcription factor AP-1	P05412	1FOS
NF-κB	Nuclear factor NF-kappa-B	P19838	3GUT
ERK	ERK-1	P27361	2ZOQ
COX-2	COX-2	P35354	3LN1^*^
JNK	c-Jun N-terminal kinase	P45983	3PZE
MKK4/7	mitogen-activated protein kinase kinase 4	P45985	3ALN
MKK3/6	mitogen-activated protein kinase kinase 6	P52564	3FME
p38	p38 MAP kinase	P53778	1CM8
MEK1/2	mitogen-activated protein kinase kinase 1	Q02750	3DY7
TRAF6:RIP1	RIP1	Q13546	4ITJ
TRAF6	TNF receptor-associated factor 6	Q9Y4K3	1LB5

^*^The structure of COX-2 was modeled by computer homology modeling based on the structure of *Mus musculus* (PDB: 3LN1) by SWISS-MODEL [48], since there was no human structure available and the identities between the two proteins from human and *Mus musculus* was 87 %

### Calculation of network efficiency and network flux

According to Michaelis-Menten equation and the law of mass action, the rate equation in presence of 1 non-competitive inhibitor I was:

1 $$ v={v}_{max}\kern0.5em \times \kern0.5em \frac{1}{\left(1\kern0.5em +\kern0.5em \frac{K_m}{\left[\mathrm{S}\right]}\right)\left(1\kern0.5em +\kern0.5em \frac{\left[\mathrm{I}\right]}{K_I}\right)}\kern0.5em =\kern0.5em {v}_o\kern0.5em \times \kern0.5em \frac{1}{\left(1\kern0.5em +\kern0.5em \frac{\left[\mathrm{I}\right]}{K_I}\right)} $$

where *K*_*m*_, *K*_*I*_, [S] and [I] are Michaelis constant, inhibition constant of I, concentration of substrate S and inhibitor I; *v*_*0*_ is the activity of the enzyme without inhibitor [45]. Thus we defined the fraction of affection (*f*_*a*_) to quantify what the percentage the enzyme was inhibited:

2 $$ {f}_a\kern0.5em =\kern0.5em 1\kern0.5em -\kern0.5em \frac{v}{v_o}\kern0.5em =\kern0.5em 1\kern0.5em -\kern0.5em \frac{1}{1\kern0.5em +\kern0.5em \frac{\left[\mathrm{I}\right]}{K_I}} $$

When 2 mutually exclusive inhibitors (X and Y) both existed in the system, *f*_*a*_ would be [45]:

3 $$ {f}_{\kern0.5em a}=\kern0.5em 1\kern0.5em -\kern0.5em \frac{v}{v_o}\kern0.5em =\kern0.5em 1\kern0.5em -\kern0.5em \frac{1}{1+\frac{\left[{\mathrm{I}}_X\right]}{K_X}+\frac{\left[{\mathrm{I}}_Y\right]}{K_Y}} $$

In the pathway network, the value of an edge (*EV*) represented the resistance in signal transduction. That is, when a target protein was inhibited, the value of the edge which came out from the target protein would enlarge to accommodate it. We arbitrarily set the initial (default) *EV* for each edge as 1. We arbitrarily assumed that the most potent inhibitor can block the target 99.5 %, thus we defined the highest *EV* as 200 (1/(1–99.5 %)). Accordingly, the *EV* at different concentration of inhibitor I would be:

4 $$ EV\kern0.5em =\kern1em {10}^{2.303\kern0.5em \times \kern0.5em {f}_a}\kern0.5em =\kern0.5em {10}^{2.303\kern0.5em \times \kern0.5em \left(1-\frac{1}{1\kern0.5em +\kern0.5em \frac{\left[\mathrm{I}\right]}{K_I}}\right)} $$

or

5 $$ EV={10}^{2.303\kern0.5em \times \kern0.5em \left(1\kern0.5em -\kern0.5em \frac{1}{1\kern0.5em +\kern0.5em \frac{\left[{\mathrm{I}}_X\right]}{K_X}\kern0.5em +\kern0.5em \frac{\left[{\mathrm{I}}_Y\right]}{K_Y}}\right)} $$

when 2 inhibitors both existed.

Network efficiency and network flux were both measures of the network connectivity [5, 18, 26]. *NE* was defined as the sum of the reciprocals of the shortest path lengths between all pairs of nodes in the pathway network:

6 $$ NE={\displaystyle {\sum}_{i\ne j\in G}\frac{1}{d_{ij}}} $$

*NF* was defined as the sum of the reciprocals of the shortest path lengths between other nodes and the exit of the pathway network:

7 $$ NF={\displaystyle {\sum}_{i\ne j\in G,\ j= exit}\frac{1}{d_{ij}}} $$

where *d*_*ij*_ is the length of the shortest path between nodes $i$ and $j$. The calculation programs of *NE* and *NF* were written in C++ language using the Dijkstra algorithm.

In order to evaluate the influence of a compound on the pathway network, we defined the *NEd* as the degree of decrease of *NE* as following:

8 $$ NEd\kern0.5em =\kern0.5em \frac{N{E}_{max}\kern0.5em -\kern0.5em NE}{N{E}_{max}\kern0.5em -\kern0.5em N{E}_{min}}\kern0.5em \times \kern0.5em 100\% $$

where *NE*_*max*_ and *NE*_*min*_ are the maximal and minimal *NE* when all *EV*s are set as 1 and 200, respectively. Similarly, the *NFd* was defined accordingly:

9 $$ NFd\kern0.5em =\kern0.5em \frac{N{F}_{max}\kern0.5em -\kern0.5em NF}{N{F}_{max}\kern0.5em -\kern0.5em N{F}_{min}}\kern0.5em \times \kern0.5em 100\% $$

Finally, we defined the *NEF* as the geometric mean of *NEd* and *NFd* to evaluate the impact of a compound on the pathway network comprehensively:

10 $$ NEF\kern0.5em =\kern0.5em \sqrt{NEd\kern0.5em \times \kern0.5em NFd} $$

### Fitting of dose–response curve

Typically, the dose–response relation can be simulated by the following equation [46]:

11 $$ \mathrm{y}\kern0.5em =\kern0.5em {E}_{max}\kern0.5em -\kern0.5em \frac{E_{max}\kern0.5em -\kern0.5em {E}_{min}}{1\kern0.5em +\kern0.5em {\left(\frac{\left[\mathrm{I}\right]}{E{C}_{50}}\right)}^n} $$

where E_max_ and E_min_ are the top and bottom asymptotes of the response, y is the inhibition rate when the concentration of the inhibitor is [I], EC50 is the concentration of inhibitor at half-maximal effect, and n is the slope parameter like the Hill coefficient [47]. The fitting of computational efficacy or experimental results *versus* the concentration of the inhibitor was performed and the correlation coefficient was used to evaluate the reliability of the model.

### Combination index

The effect of the combination of 2 drugs may be simple additive, exaggerated (synergistic) or attenuated (antagonistic). In order to quantify the synergism or antagonism for 2 drugs (D_1_ and D_2_), Chou T. C. introduced the term *combination index* [13]:

12 $$ \mathrm{C}\mathrm{I}\kern0.5em =\kern0.5em \frac{(D)_1}{{\left({D}_x\right)}_1}\kern0.5em +\kern0.5em \frac{(D)_2}{{\left({D}_x\right)}_2} $$

where *CI* <1, =1 and >1 indicated synergism, additive effect and antagonism, respectively. (D_x_)_1_ and (Dx)_2_ represented the concentrations when D_1_ and D_2_ alone can inhibit the system x %. (D)_1_ and (D)_2_ were the concentrations when D_1_ and D_2_ in combination can inhibit the system x %.

### Experimental

#### RAW264.7 Cell experiments

All compounds for *in intro* test were purchased from National Institute for Food and Drug Control (Beijing, China). Lipopolysaccharide (LPS) was purchased from Nanjing Baikang Biological Technology Co., Ltd. (Nanjing PR China). The inhibitory activities of compounds against LPS-induced PGE_2_ production were determined in RAW246.7 cells (Cell Culture Center of the Chinese Academy of Medical Sciences, Beijing, China). First, RAW246.7 cells were cultured in high-glucose Dulbecco’s Modified Eagle’s medium (DMEM, Gibco, Carlsbad, USA) which contained streptomycin (100 μg ml^−1^), penicillin (100 U ml^−1^) and 10 % (v/v) fetal bovine serum (FBS, Sijiqing, Deqing, Hangzhou, China) at 37 °C in a humidified incubator containing 5 % CO_2_. Second, the cell viability was determined by MTT assays to evaluate the cellular toxicity of compounds. RAW246.7 cells were plated in 96-well plates (4 × 10^4^ cells/well) overnight and treated 24 h with various concentrations of compounds in FBS-free DMEM. MTT (5 mg mL^−1^) was added in each well and the cells were incubated for 4 h at 37 °C. Then the standard protocol of MTT assays was adopted to determine the cell viability by SpectraMax M2e Microplate Reader (Molecular Devices, Menlo Park, USA). Third, RAW264.7 cells were pretreated with various concentrations of compounds or positive drug (Celecoxib) for 1 h. Then LPS (final concentration 1 μg ml^−1^) was added and the cells were incubated for 16 ~ 18 h. The concentration of PGE2 was measured by Prostaglandin E_2_ EIA kit (Enzo Life Sciences, Farmingdale, NY, USA). The experiments were repeated 3 times at each concentration of each compound. The inhibition rate of a compound against LPS-induced PGE_2_ production was calculated by:

13 $$ Inhibition\  rate\kern0.5em =\kern0.5em \left(1\kern0.5em -\kern0.5em \frac{C\left( drug\kern0.5em -\kern0.5em C(control)\right)}{C(model)\kern0.5em -\kern0.5em C(control)}\right)\kern0.5em \times \kern0.5em 100\% $$

where *C*(control) was the background concentrations of PGE2. *C*(drug) and *C* (model) represented the concentrations of PGE2 when the RAW246.7 cells were incubated with drug or DMSO and then stimulated by LPS, respectively.

## Conclusions

In this study, we developed a quantitative model to predict the dose–response curves of single drug and drug combination based on the pathway network of LPS-induced PGE2 production. The network efficiency and network flux are both measures of the connectivity of the pathway network. And thus the degrees of the decrease of *NE*, *NF* and *NEF* could evaluate the efficacy of a drug to the biological systems. By integrating molecular docking and network analysis, the dose–response relationships of 5 compounds against LPS-induced PGE2 production were evaluated and the predictions agreed well with experimental results. Furthermore we explored the dose–response relationships of drug combinations to study the synergism. Moreover, identifying novel effective drug combinations or multi-target agents is a new trend in drug discovery. Actually, the herb medicines are the natural combinations of active compounds. This computational method can be used to quantitatively evaluate the efficacy of a mixture of 2 or more drugs, even herb medicines. The increasing of the complexity of multiple omics data sets requires more effective approaches for drug screening. And computational modeling is an indispensable tool for understanding dose–response relationship and mechanisms of a drug or drug combination. This work would provide a new computational approach to evaluate drug efficacy before clinic trials and screen optimal combination for drug discovery when a biological system/process has a well-defined pathway.

## Abbreviations

Caa: Caffeic acid
CI: *Combination index*
DRS: Dose–response surface
LPS: Lipopolysaccharide
NE: Network efficiency
NEF: The geometric mean of the degree of decrease of network efficiency and the degree of decrease of network flux
NF: Network flux
PGE2: Prostaglandin E2
Sco: Scopoletin
